# Association of admission hyperglycemia and all-cause mortality in acute myocardial infarction with percutaneous coronary intervention: A dose–response meta-analysis

**DOI:** 10.3389/fcvm.2022.932716

**Published:** 2022-09-12

**Authors:** Shao-Yong Cheng, Hao Wang, Shi-Hua Lin, Jin-Hui Wen, Ling-Ling Ma, Xiao-Ce Dai

**Affiliations:** ^1^Department of Cardiology, Beijing Royal Integrative Medicine Hospital, Beijing, China; ^2^Department of Internal Medicine, Zhejiang Hospital, Hangzhou, China; ^3^Department of Endocrinology and Metabolism, Affiliated Hospital of Chengdu University, Chengdu, China; ^4^Department of Cardiology, Affiliated Hospital of Jiaxing University, Jiaxing, China

**Keywords:** admission hyperglycemia, mortality, acute myocardial infarction, percutaneous coronary intervention, MACE

## Abstract

**Objective:**

The aim of this study is to evaluate the associations between admission hyperglycemia and the risk of all-cause mortality in patients with acute myocardial infarction (AMI) with or without diabetes, to find optimal admission glucose intervention cut-offs, and to clarify the shape of the dose–response relations.

**Methods:**

Medline/PubMed and EMBASE were searched from inception to 1 April 2022. Cohort studies reporting estimates of all-cause mortality risk in patients with admission hyperglycemia with AMI were included. The outcomes of interest include mortality and major adverse cardiac events (MACEs). A random effect dose–response meta-analysis was conducted to access linear trend estimations. A one-stage linear mixed effect meta-analysis was used for estimating dose–response curves. Relative risks and 95% confidence intervals were pooled using a random-effects model.

**Results:**

Of 1,222 studies screened, 47 full texts were fully reviewed for eligibility. The final analyses consisted of 23 cohort studies with 47,177 participants. In short-term follow-up, admission hyperglycemia was associated with an increased risk of all-cause mortality (relative risk: 3.12, 95% confidence interval 2.42–4.02) and MACEs (2.34, 1.77–3.09). In long-term follow-up, admission hyperglycemia was associated with an increased risk of all-cause mortality (1.97, 1.61–2.41) and MACEs (1.95, 1.21–3.14). A linear dose–response association was found between admission hyperglycemia and the risk of all-cause mortality in patients with or without diabetes.

**Conclusion:**

Admission hyperglycemia was significantly associated with higher all-cause mortality risk and rates of MACEs. However, the association between admission hyperglycemia and long-term mortality risk needs to be determined with caution. Compared with current guidelines recommendations, a lower intervention cut-off and more stringent targets for admission hyperglycemia may be appropriate.

**Systematic review registration:**

[https://www.crd.york.ac.uk/prospero/display_record.php?ID=CRD42022317280], identifier [CRD42022317280].

## Introduction

Acute myocardial infarction (AMI) is a common form of coronary heart disease with high mortality. The annual incidence of AMI in the United States is estimated at 605,000 and has continued to decline over the past few decades (1). Although the improvement of cardiovascular disease prevention and control has led to a downward trend in the incidence of AMI (2), there are still other risk factors affecting the mortality of AMI. Admission hyperglycemia (AH) has been reported to negatively affect AMI. Two previous meta-analyses assessed the association of AH with all-cause mortality in patients with AMI (3, 4). The authors included 27 studies in two AMI-related treatment eras (thrombolytic therapy and percutaneous coronary intervention) and the results indicated that AH was associated with higher all-cause mortality and complications such as heart failure and cardiac shock in AMI regardless of diabetes mellitus (DM) or revascularization treatments. During short-term (in-hospital and 30-day) follow-up, patients with AH without DM had a 4-fold higher risk of all-cause mortality than normoglycemic patients, which was two times higher than patients with DM with AH. Although these two meta-analyses seemed to describe the same trend of AH, they did not clarify whether we need to pay more attention to patients with non-DM with AH because these patients had a higher risk of mortality. The treatment target range of admission glucose in patients with AMI is still uncertain.

According to the clinical practice of diabetes, AH was defined as blood glucose levels > 140 mg/dl (7.8 mmol/L) (5); however, the definition of AH in patients with AMI varied from study to study because some studies used the different thresholds of AH such as the DM diagnosis or self-defined criteria (6–8). Therefore, when using the AH as a marker of impaired prognosis, an inaccurate assessment of the risk of all-cause mortality may occur. Based on available evidence, assessment and monitoring of glycemic status in all patients with AMI are recommended (9, 10). When AH is higher than 200 mg/dl (11 mmol/L), it is reasonable for patients with ST-segment elevation myocardial infarction (STEMI) to have less stringent glycemic control. Additionally, the guideline of NSTE-ACS (10) suggests that patients need to lower their glucose when AH is more than 180 mg/dl (10 mmol/L) but the most important limitation of this recommendation is that it only reflects some critically ill patients (11) and this evidence comes from out-of-date studies (12, 13) that have been published more than two decades. To date, there are still no reliable data to guide optimal glucose management.

Given that no meta-analysis has assessed the association between risk of all-cause mortality and mild-to-severe AH in AMI and suggested whether the risk of all-cause mortality is the same in patients without DM AH or in patients with DM AH, we hypothesize that mild-to-severe AH is associated with a different risk of all-cause mortality in patients with AMI and that the risk is different in patients with or without DM. We aimed to perform a systematic review and dose–response meta-analysis to investigate the association of AH levels with the risk of all-cause mortality in AMI patients with or without DM and to find the optimal target range of intervention for admission glucose. Whenever possible, we clarified the shape of the dose–response relations.

## Methods

This meta-analysis was conducted in accordance with the Preferred Reporting Items for Systematic reviews and Meta-Analyses (PRISMA) guideline (14). The protocol was registered with the PROSPERO (CRD42022317280).

### Data sources and searches

We carried out systematic electronic searches, without language restriction, in Medline/PubMed and EMBASE for relevant literature from database inception to 1 April 2022. Electronic search was conducted with controlled vocabulary (MeSH in PubMed and Emtree in EMBASE) and keywords as search terms. The search terms included *hyperglycemia, myocardial infarction, blood glucose, plasma glucose, primary angioplasty, and percutaneous coronary intervention*. The full search strategy is available in Supplementary File 1. Three reviewers (S.-Y. C., H.W., and X.-C. D.) independently screened titles and abstracts. In addition, we used backward snowballing (i.e., a review of references from identified articles and pertinent reviews).

### Eligibility and study selection

Studies were considered eligible if they evaluated the association between AH and all-cause mortality in patients with AMI with primary percutaneous coronary intervention (PPCI) or within 24 h of PCI. The title and abstract of all articles were reviewed by two investigators (S.-Y. C., and H.W.). They selected potentially eligible studies that (1) were conducted in adults (aged > 18 years); (2) had a cohort-study design; (3) had a sample size of more than 100 participants; (4) considered hyperglycemia as exposure and in at least two quantitative categories; (5) considered all-cause mortality as the outcome of interest; (6) reported adjusted or unadjusted odds ratio (OR) or relative risk (RR) or hazard risk (HR) across categories of exposures or provided the total number of participants and events across categories of exposures. For duplicate publications, those with the largest number of participants were included. For these studies, the publication with the categorical model was included in dose–response analyses. We excluded studies if the study was a case-control study, an exposure group receiving thrombolysis, or opting for conservative treatment, and no clear classification interval was given.

### Data extraction and assessment for study quality

Data were extracted independently by two investigators (S.-Y. C. and X.-C. D.) using a standardized form in Word (Microsoft Corporation). Extracted data were checked independently by two investigators (H.W. and X.-C. D.). The following information was extracted from each study: author, year, country or region, population, glucose measurement, hyperglycemia category, sample size, proportion of hyperglycemia individuals, follow-up duration, confounding factors in the multivariable analysis, and outcomes. The AH categories were defined as those in their own study criteria. We reported outcomes at short (in-hospital or up to 90 days) and long (no less than 1 year)-term duration. We used the maximally adjusted effect size reported in studies. When studies controlled classic cardiovascular disease risk factors, such as hypertension, lipid profile, diabetes, sex, and smoking, we primarily selected this model in our meta-analysis. We performed subgroup analyses based on follow-up duration, diabetic status, type of AMI, time to PCI, geographical region, adequate adjustment, study quality, and glucose level assessment. Newcastle–Ottawa Scale (NOS) was used to assess the quality of included studies in this meta-analysis (15). Three domains (cohort selection, comparability, and outcome) were independently assessed by three investigators (J.-H. W., H.W., and X.-C. D.). The maximum score of NOS was 9 which meant the highest quality. According to the NOS manual, we upgraded the score if participants were from the same communities; studies reported the important confounders; studies reported important factors (e.g., sex, hypertension, smoking, and diabetes); studies reported outcomes clearly or follow-up duration no less than 1 year. Inconsistency was resolved through discussion.

### Outcomes

The primary outcomes of this meta-analysis were short- and long-term all-cause mortality. For short-term mortality, we mainly used in-hospital all-cause mortality. If in-hospital mortality was not reported, we used 30–90-day mortality. The definition of the duration of long-term follow-up varied by study, but we defined it as three groups: 1 year, 1–3 years, and follow-up beyond 3 years. We chose this approach to maximize the number of studies included in our primary analysis. The secondary outcomes were defined as major adverse cardiac events (MACEs), namely, cardiac death, heart failure, reinfarction, target vessel revascularization, and stroke.

### Data synthesis and statistical analysis

A descriptive analysis, AH category, and follow-up of each study are shown in Supplementary File 2. Pooled RRs and their corresponding 95% confidence interval (CI) for categorical variables (dichotomous outcomes) were calculated using a random effect model to minimize the effect of clinical and methodological heterogeneity among studies, with the inverse variance method. We calculated adjusted or unadjusted RRs with 95% CIs for the overall effect estimate. When an HR was reported, we considered it equivalent to the relative effect measure reported in other studies. Adjusted odd ratios were considered equal to adjusted RRs when disease incidence was low (< 10%). If not, convert OR to RR using the following equation: (RR = OR/[(1- pRef) + (OR × pRef)]), where pRef is the prevalence of the outcome in the non-exposed group (16). We attempted to establish a dose–response relationship between AH and all-cause mortality risk in AMI through the robust-error meta-regression (REMR) model (17). This is a one-stage method that considers all included studies as a whole while treating each study as a cluster in order to validate the fitting of the dose–response curve. Generally, there are three possible dose–response gradients in epidemiological studies: linear, non-linear, and categorical dose–response relationship (18). The last one is not as precise as the former two (19). A linear or non-linear relationship could be established when there are sufficient dose-specific points, say, 10 points for the linear and 15 points for the non-linear relationship (20). In order to consider both linearity and non-linearity in one model, we used the restricted cubic spline (RCS) function to fit the dose–response trend. Three random knots were used in the RCS function (20). These three knots divided the data into four pieces and the dose–response curve was fitted within each piece and further smoothed at the knots. The first and last pieces will be restricted to linear, while for the second and third pieces, a cubic spline was fitted. When the cubic and quadratic terms in the function are equal to zero, RCS automatically degrades into a simple linear function. The Wald test was used to test the probability that the cubic and quadratic terms were equal to zero (21). The median point in each category was assigned to the corresponding RR. When studies did not report the direct median of each category, we estimated approximate medians by using the midpoint of the lower and upper bounds. We use the lower bound divided by 1.2 or the upper bound multiplied by 1.5 when no median is reported for the open-ended category. When studies reported separate effect sizes across sex or other subgroups (follow-up duration, diabetic status, type of AMI, time to PCI, geographical region, adequate adjustment, study quality, and glucose level assessment), we pooled the specific estimated by a fixed-effect model. Because the definition of glycaemia was different among studies, when studies did not consider the lowest category as the reference, we excluded the categories below the reference category to measure the dose–response relations.

We evaluated the potential influence of each study on the summary RRs by re-estimating the RRs after omitting one study at a time. We tried to perform subgroup analyses by sex, geographical location, glycaemia assessment method, follow-up duration, stress-related factor, and type 2 diabetes. *I*^2^ values were used to estimate statistical heterogeneity (22). *I*^2^ < 25%, 50%, or > 50% indicated mild, moderate, or substantial heterogeneity, respectively (22). Publication bias was assessed by the visual inspection of a contour-enhanced funnel plot (23), and examined by Egger’s test (24); Begg’s test (25) was also assessed if the number of included studies exceeded 10.

Some studies did not consider the lowest category as the reference; therefore, we recalculated effect sizes by assuming the lowest category as the reference if it was not the hypoglycemia group. All statistical analyses were conducted in STATA (Stata Version 16.0; Stata Corp.). Comparisons were two-tailed using a threshold of *P* ≤ 0.05 for statistical significance for all analyses.

### Assessment of evidence certainty

The Grading of Recommendations Assessment, Development, and Evaluation (GRADE) was used to summarize the quality of evidence on an outcome-by-outcome basis graded as high, moderate, low, or very low (26). We did not rate down the strength of evidence for the risk of bias if the subgroup analysis showed no association of admission glucose level effects with the risk of bias. The GRADE Profiler (Windows-only tool, GRADEpro) was used to construct summary tables.

## Results

### Studies retrieved and characteristics

**FIGURE 1:**
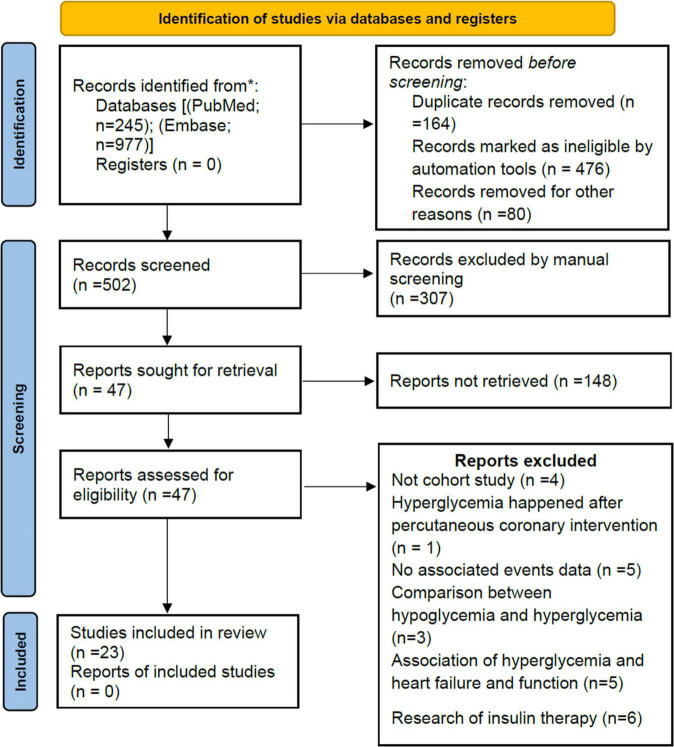
PRISMA flow diagram.

Our initial search returned 1,222 results. After the title and abstract, 47 articles were considered potentially relevant. Twenty-three studies were included after a full-text review (Figure 1). Ten studies (8, 27–35) reported short-term all-cause mortality, four studies (36–39) reported long-term all-cause mortality, and nine studies (6, 7, 40–46) reported both short- and long-term all-cause mortality, which were included in the primary meta-analysis. Eight studies (7, 29–31, 34, 37, 43, 45) were also used for secondary outcomes.

In total, 47,177 participants were enrolled, including 28,369 participants (60.1%) with AH. Follow-up in most studies ranged from 1 to 6 years. The publication year ranged from 2005 to 2021 (Supplementary File 2). Of the 23 studies included, 13 were from Asia (Israel, Japan, Turkey, China, Indonesia, Korea, and Pakistan), six from Europe (Switzerland, Italy, Portugal, and the Netherlands), three from North America (Canada and United States), and one from Africa (Egypt). Based on adjusted confounders, seven studies met our criteria for adequate adjustment, while 16 studies were insufficiently adjusted for potential confounders (Supplementary File 2). All but one study (fair quality) was rated as good quality according to the NOS quality assessment. Details of the assessment are shown in Supplementary File 3.

### Short-term follow-up outcomes

#### All-cause mortality

**FIGURE 2:**
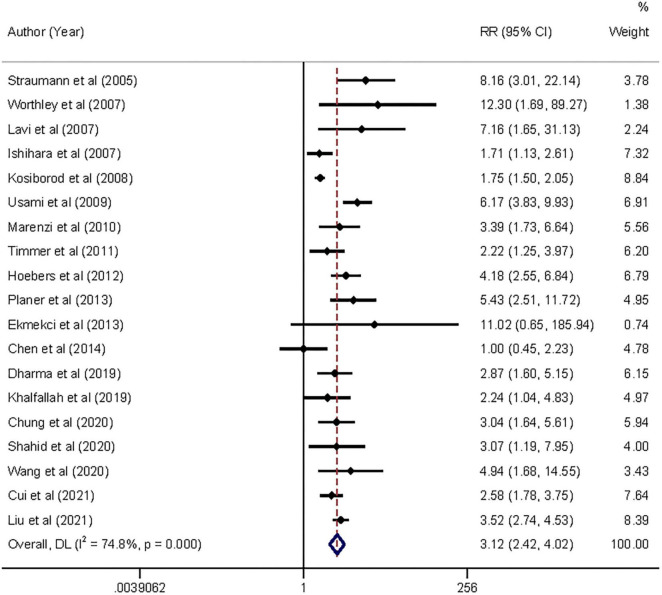
Forrest plot of admission hyperglycemia associated with the short-term all-cause mortality risk. Weights are from random-effects model.

Moderate-quality evidence from 19 cohort studies, including all-cause mortality in 2,380 of 39,629 participants, showed an association between AH and short-term risk of all-cause mortality in patients with AMI regardless of diabetes. The pooled analysis indicated that admission hyperglycemia was associated with a 2.12-fold increased risk of all-cause mortality: relative risk 3.12 (95% confidence interval 2.42–4.02) with substantial heterogeneity (*I*^2^ = 74.8%, *P*_*heterogeneity*_ < 0.001; Figure 2). The relative risk did not materially alter the results (relative risk range 2.93–3.29) when each study was sequentially omitted from the main analysis (Supplementary File 4A).

In the exploratory subgroup analysis, AH was associated with an increased risk of all-cause mortality (relative risk range 1.85–3.55; Table 1). A trend was found toward a higher risk with increasing follow-up duration. The association was significant when we controlled for geographical regions and strengthened when we controlled for type of AMI: relative risk 3.54 (2.86–4.38, *I*^2^ = 54.7, *n* = 12) in STEMI and 3.52 (2.74–4.53, *I*^2^ = 0, *n* = 1) in NSTEMI. Subgroup analyses suggested that geographic regions and AMI type were potential sources of heterogeneity. Publication bias was found in Egger’s test (*P* = 0.015) but not in Begg’s test (*P* = 0.327). In the contour-enhanced funnel plot (Supplementary File 5), the results suggested that non-reporting bias might exist.

**TABLE 1:** Subgroup analysis of association between admission hyperglycemia and short-term outcomes in patients with AMI.

Characteristics	*n*	Relative risk (95%CI)	*I*^2^(%)	P for interaction
**All-cause mortality**				
All studies	19	3.12 (2.42–4.02)	74.8	–
**Follow-up duration**				
In-hospital	13	2.43 (2.18–2.72)	79.3	0.51
30–90 days	6	2.66 (2.10–3.36)	61.8	
**Diabetes mellitus**				
Yes	13	2.30 (2.02–2.62)	0	0.36
No	15	2.12 (1.88–2.39)	88.2	
**Type of AMI**				
STEMI	12	3.54 (2.86–4.38)	54.7	< 0.001
NSTEMI	1	3.52 (2.74–4.53)	0	
STEMI or NSTEMI	6	1.97 (1.73–2.24)	70.4	
**Time to PCI**				
< 2 h	13	2.73 (2.24–3.32)	49.9	0.26
Up to 24 h	6	2.39 (2.12–2.69)	89.2	
**Geographical region**				
Asia	11	3.04 (2.61–3.55)	64.4	< 0.001
Europe	4	3.55 (2.60–4.85)	46.9	
North America	3	1.85 (1.59–2.16)	82.6	
Africa	1	2.24 (1.04–4.83)	0	
**Adequate adjustments**				
Yes	6	3.04 (2.34–3.95)	65.2	0.10
No	13	2.39 (2.14–2.66)	77.9	
**Study quality**				
Good	18	2.47 (2.23–2.73)		0.65
Fair	1	3.07 (1.19–7.94)	0	
**Glucose level assessment**				
Random plasma glucose	16	2.44 (2.19–2.71)	77.9	0.33
Fasting plasma glucose	3	2.91 (2.07–4.10)	27.7	
**MACEs**				
All studies	10	2.34 (1.77–3.09)	84.2	–
**Follow-up duration**				
In-hospital duration	7	2.08 (1.81–2.38)	58.9	0.019
30–90 days	3	2.75 (2.27–3.34)	94.3	
**Diabetes mellitus**				
Yes	5	2.29 (1.90–2.76)	68.2	0.677
No	9	2.18 (1.94–2.46)	86.8	
**Type of AMI**				
STEMI	8	2.30 (2.03–2.60)	87.6	0.844
NSTEMI	1	2.09 (1.49–2.92)	0	
STEMI or NSTEMI	1	2.38 (1.67–3.40)	0	
**Time to PCI**				
< 2 h	7	2.07 (1.81–2.37)	68.8	0.017
Up to 24 h	3	2.78 (2.28–3.40)	92	
**Geographical region**				
Asia	7	2.30 (2–2.65)	88.1	0.107
Europe	1	2.30 (1.71–3.09)	0	
North America	1	2.80 (1.95–4.03)	0	
Africa	1	1.67 (1.26–2.19)	0	
**Glucose level assessment**				
Random blood glucose	7	2.31 (2.02–2.63)	89.1	0.728
Fasting blood glucose	3	2.21 (1.80–2.71)	0	

**FIGURE 3:**
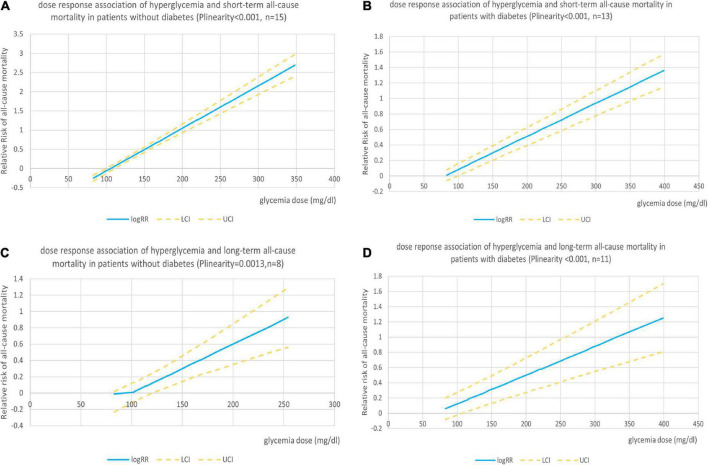
**(A)** Dose–response association of admission hyperglycemia with short-term risk of all-cause mortality in patients with AMI without diabetes. **(B)** Dose–response association of admission hyperglycemia with short-term risk of all-cause mortality in patients with AMI with diabetes. **(C)** Dose–response association of admission hyperglycemia with long-term risk of all-cause mortality in patients with AMI without diabetes. **(D)** Dose–response association of admission hyperglycemia with long-term risk of all-cause mortality in patients with AMI with diabetes.

Nineteen studies reported sufficient data for the dose–response analyses. We found no evidence of the non-linear association between admission hyperglycemia and all-cause mortality in patients with and without diabetes (P_*non–linearity*_ = 0.302 and 0.194, respectively), so the linear dose–response effect was analyzed. The analysis showed significant dose-dependent relations between admission hyperglycemia and mortality risk in patients with non-DM and DM (both P_*linearity*_ < 0.001, *n* = 15 and 13, respectively, Figures 3A,B). In patients with non-DM, the risk of all-cause mortality increased by 20% when every 18 mg/dl (1 mmol/l) dose of glucose level was increased (relative risk 1.20; 95% confidence interval 1.18–1.22). In patients with DM, the risk of all-cause mortality increased by 8% when every18 mg/dl (1 mmol/l) dose of glucose level was elevated (relative risk: 1.08; 1.07–1.09).

#### Major adverse cardiac events

Low-quality evidence from 10 cohort studies with 11,520 participants and 1,791 MACEs events performed the analysis of admission hyperglycemia and the risk of MACEs in patients with AMI regardless of diabetes. Admission hyperglycemia was associated with 1.34-fold higher risk: relative risk 2.34 (95% confidence interval 1.77–3.09, *I*^2^ = 84.2%, *n* = 10; Supplementary File 6). The relative risk did not materially alter the results (relative risk range 2.07–2.54) when we omitted each study one at a time (Supplementary File 4B). In the subgroup analysis, AH was associated with an increased risk of MACEs (relative risk range 1.67–2.80; Table 1). An association became stronger in North America than in Asia or Europe (2.8 vs. 2.30) but became weaker in Africa: 1.76 (1.26–2.19). When follow-up duration increased, the association became stronger. The subgroup analyses suggested that follow-up duration, time to PCI, and geographic regions were potential sources of heterogeneity. No evidence of publication bias was observed in Egger’s test (*P* = 0.549) and Begg’s test (*P* = 0.152).

### Long-term follow-up outcomes

#### All-cause mortality

**FIGURE 4:**
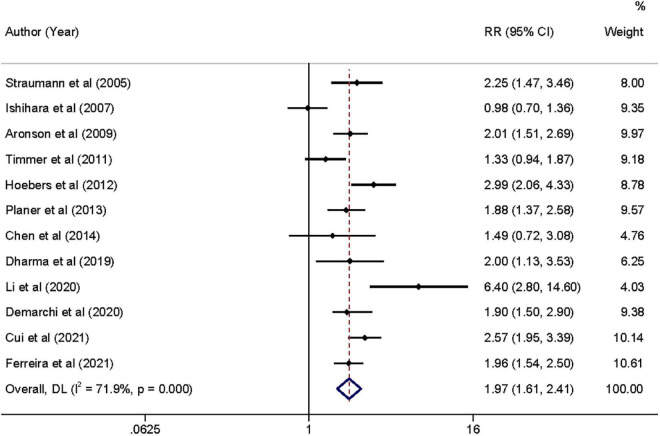
Forrest plot of admission hyperglycemia associated with the long-term all-cause mortality risk. Weights are from random-effects model.

Moderate-quality evidence from 12 studies showed risk estimates of all-cause mortality in relation to long-term follow-up duration (at least 1 year). We observed a 0.97-fold higher risk of total mortality associated with admission hyperglycemia in the analysis of all participants: relative risk 1.97 (95% confidence interval 1.61–2.41, *I*^2^ = 71.9%; Figure 4). The exclusion of each of one study at one time did not change the results (Supplementary File 4C). During the long-term follow-up, the association was more significant in patients with non-DM than patients with DM (2.12 vs. 1.73; Table 2). Correlation became stronger in patients who underwent PCI within 24 h compared to primary PCI (< 2 h; 2.16 vs. 1.74). Time to PCI and DM status was potential sources of heterogeneity. No evidence was found of publication bias in Egger’s test (*P* = 0.563) and Begg’s test (*P* = 0.945).

**TABLE 2:** Subgroup analysis of association between admission hyperglycemia and long-term outcomes in patients with AMI.

Characteristics	*n*	Relative risk (95%CI)	*I*^2^(%)	P for interaction
**All-cause mortality**				
All studies	12	1.97 (1.61–2.41)	71.9	–
**Median follow-up duration**				
1 year	3	1.66 (1.33–2.06)	24.6	0.289
1–3 years	6	2.03 (1.77–2.33)	84.9	
> 3 years	3	1.98 (1.62–2.42)	0	
**Diabetes mellitus**				
Yes	11	1.73 (1.55–1.93)	34	0.018
No	8	2.12 (1.86–2.43)	58.5	
**Type of AMI**				
STEMI	8	1.88 (1.63–2.15)	65.7	0.349
NSTEMI	1	2.28 (1.79–2.90)	0	
STEMI or NSTEMI	4	1.88 (1.60–2.20)	85.4	
**Time to PCI**				
< 2 h	8	1.74 (1.51–2.01)	78.4	0.038
Up to 24 h	4	2.16 (1.87–2.50)	0	
**Geographical region**				
Asia	6	1.90 (1.63–2.23)	82.6	0.937
Europe	5	1.97 (1.70–2.28)	61.1	
North America	1	1.88 (1.37–2.58)	0	
**Adequate adjustments**				
Yes	5	1.82 (1.58–2.09)	80.7	0.202
No	7	2.08 (1.79–2.41)	64.4	
**Glucose level assessment**				
Random blood glucose	10	1.83 (1.62–2.05)	73.6	0.058
Fasting blood glucose	2	2.28 (1.87–2.79)	31.1	

One study (45) did not report sufficient data, so 11 studies were included in the dose–response analysis. We found no evidence of the non-linear association between admission hyperglycemia and long-term follow-up all-cause mortality in patients with or without DM (P_*non–linearity*_ = 0.102 and 0.263, respectively). We found a linear positive correlation between the dose of glycaemia level and risk of all-cause mortality in patients with non-DM (P_*linearity*_ = 0.001, *n* = 8; Figure 3C) and patients with DM (P_*linearity*_ < 0.001, *n* = 11; Figure 3D). The results showed that the risk of all-cause mortality in patients with DM increased by 7% when every 18 mg/dl (1 mmol/l) dose of glucose level was elevated (relative risk 1.07; 1.04–1.09) and by 11% (RR: 1.1; 1.06–1.16) in patients with non-DM.

### Major adverse cardiac events

Very low-quality evidence from four studies with 5,942 participants and 1,229 MACEs events suggested that admission hyperglycemia had an association with the risk of MACEs. Admission hyperglycemia was associated a 95% higher risk: relative risk 1.95 (95% confidence interval 1.21–3.14, *I*^2^ = 89.3%, *n* = 4; Supplementary File 7). Exclusion of each of one study at one time did not materially alter the results (relative risk range 1.95–2.38), except for one study (37) which changed the results (1.96; 0.86–4.48; Supplementary File 4D). Because the number of included studies was limited, we did not perform subgroup analyses. No evidence was found of publication bias in Egger’s test (*P* = 0.301).

## Discussion

### Principal findings

In this systematic review and dose–response meta-analysis, we pooled data from 23 cohort studies to present a relatively comprehensive overview of the association of admission hyperglycemia with all-cause mortality risk in patients with AMI with or without diabetes mellitus. We found that admission hyperglycemia was significantly and positively associated with higher all-cause mortality risk in the short-term follow-up than in long-term follow-up (moderate-quality evidence, Supplementary File 8). However, in our exploratory subgroup analysis, patients with AMI with DM had lower all-cause mortality than patients without DM with increasing follow-up. Compared with patients with non-DM, admission plasma glucose levels were generally higher in patients with DM. We found that the association remained significant after DM was controlled, which indicated that admission hyperglycemia was an independent marker of all-cause mortality risk. We observed that admission hyperglycemia was significantly associated with the risk of MACEs no matter what the follow-up duration (low to very low-quality evidence, Supplementary File 8). In dose–response analyses, linear associations were found between the risk of all-cause mortality and elevated plasma glucose levels at short- and long-term follow-up and the results showed that compared with patients with DM, the risk of mortality was increased faster in patients without previous DM.

### Potential mechanisms

Increased glucose levels at hospital presentation (acute hyperglycemia) are associated with numerous adverse effects leading to poor prognosis in patients with AMI regardless of diabetes mellitus (3, 47). Interestingly, patients without DM who had a mildly increased admission glucose (110–144 mg/dl) had a higher risk of mortality than patients with DM with moderately increased admission glucose (180–198 mg/dl). The incidence of AH ranged from 51 to 61.7% (with a cut-off value of 140 mg/dl) in AMI (48). The degree of oxidative stress was most strongly associated with acute rather than chronic glucose fluctuations (49). Acute hyperglycemia rapidly inhibited flow-mediated vasodilatation and may increase the production of oxygen-derived free radicals (50), which in turn can further damage the myocardium (51). Furthermore, increased oxidative stress interfered with nitric oxide-mediated vasodilatation and reduced coronary blood flow at the microvascular level. Hyperglycemia had also been shown to have lots of multiple prothrombotic effects (enhanced thrombin formation, platelet activation, and fibrin clot resistance to lysis) which might increase the risk of thrombotic complications in clinical setting (52, 53). They may be associated with reperfusion failure (no reflow) in patients with STEMI undergoing primary PCI (54). Hyperglycemia was a reflection of relative insulinopenia, which resulted in decreased glycolytic substrate for cardiac muscle and excessive free fatty acids and diminished myocardial glucose uptake in patients with AMI (3, 55). Myocardial ischemia led to an increased rate of glycogenolysis and glucose uptake through the translocation of the GLUT-4 receptor to the sarcolemma (56). Because glucose oxidation required less oxygen to generate per molecule of ATP than free fatty acid oxidation, myocardial energetics became more efficient when the dependence of ischemia on glucose oxidation was increased (57). However, due to the relative decrease in blood insulin, ischemic myocardium was forced to use free fatty acids instead of glucose as an alternative energy source because myocardial glucose uptake is acutely impaired (57). In addition, the accumulation of excess free fatty acids can lead to reduced myocardial contractility, thereby increasing the risk of pump failure and arrhythmias (55). AH also induces an increase in inflammatory cytokines with concomitant endothelial dysfunction, which leads to poorer clinical outcomes (58, 59). Therefore, in the setting of hyperglycemia and insulin resistance, a metabolic crisis may ensue as the energy efficiency of the hypoxic myocardium was reduced.

### Comparison with other studies

In our meta-analysis, at short-term follow-up, we found a 2.12-, 1.3-, and 1.12-fold higher risk of all-cause mortality associated with admission hyperglycemia in the total population, patients with and without diabetes, respectively. The results were relatively weaker than those of two previous pooled analyses of 27 studies, which suggested a 3.3-, 0.71-, and 2.93-fold higher risk for AH in short-term mortality in total population, patients with DM, and patients with non-DM, respectively (3, 4). The weaker association found in our study might be due to advances in reperfusion therapy (popularization of PCI and improvements in stent) which can shorten ischemia time and preserve more myocardial function compared with the thrombolysis era. However, in the study by Singh et al. (4), they suggested that patients still had a significantly high risk of mortality (RR 4.30) when those patients were treated with primary angioplasty. A few reasons can explain that. They found that patients with AMI with AH would have more MACEs such as cardiac shock and malignant arrhythmias which could increase the risk of mortality. What is more, some participants had prior MI or combined with multivessel coronary disease which was unavoidable risk factors that increased death for new AMI. Intraoperative no-flow phenomenon leading to reperfusion failure might be an important cause of increased mortality (54). Another reason was that the pooled results only focused on all patients with AH, which might ignore the effect caused by patients with hyperglycemic DM. Additionally, the included cohort studies were relatively monotonic (75% from Western countries). In our study, we included 14 non-Western cohort studies and provided a wider range of glucose levels; so, we reported a weaker association than in previous meta-analyses.

In the analysis of in-hospital and up 30–90 days mortality, the results were much stronger in the subgroups of studies that reported STEMI (relative risk 3.54) and NSTEMI (RR 3.52) than in subgroups that reported the composite of AMI (1.97), but only one study reported the relative risks for NSTEMI. When adequate adjustments for STEMI were controlled (43), the association between AH and mortality risk remained unchanged (RR 3.53). We found a 21% higher risk of mortality at 30–90 days than in-hospital follow-up. The definition of hyperglycemia among studies may have contributed to these results, as some studies used DM diagnosis criteria (38), some used the current admission criteria for hyperglycemia (31), and others used self-defined criteria (7, 30, 40). Since AH was positively associated with the risk of all-cause mortality, the risk of severely elevated plasma glucose concentrations was higher than mildly or moderately elevated glucose concentrations. Another explanation was that participants from different regions of the world (*P* < 0.001) might have influenced the pooled results, because most studies in Western countries used the risk of in-hospital mortality. We also found that fasting plasma glucose appeared to be a more accurate indicator of mortality risk than random plasma glucose (RR 2.91 vs. 2.44), which was supported by a previous study (38). At the time of admission, fasting plasma glucose was defined as the plasma glucose on the second day of admission when patients generally had undergone primary PCI that might reduce the norepinephrine-mediate effects (60).

In a linear dose–response meta-analysis of mortality risk in patients with and without DM, we found a 12% higher risk of death in patients with non-DM compared with patients with DM (20% vs. 8%; per 18 dl/ml: 1 mmol/l). This may be a higher risk for patients with non-DM in the early stages of elevated blood glucose levels (> 110 mg/dl). A previous study suggested that for every 18 mg/dl (1 mmol/l) increase in the glucose level there was a 4 and 6% increase in mortality in patients with non-DM and DM, respectively (61), but most patients received thrombolysis or conservative treatment. In a study of patients with STEMI undergoing primary PCI, it was shown that each 18 mg/dl (1 mmol/l) increase in the glucose level was associated with a 14% increase in mortality in patients with non-DM and a 12% increase in mortality in patients with DM (6). For the increase in the glucose level (≥ 200 mg/dl), an increase in mortality risk of 10% had been reported for patients with STEMI undergoing primary PCI (46).

In this study, we found that the risk of short-term all-cause mortality at least doubled in patients with non-DM with admission glucose concentrations > 150 mg/dl (8.33 mmol/l) and patients with diabetes with > 200 mg/dl (11.1 mmol/l). *Post hoc* analysis (43) of the HORIZONS-AMI trial showed cut-off values of 149 mg/dl and 231 mg/dl in patients with STEMI without or with diabetes, but with limited accuracy. Because patients with non-diabetes and diabetes had different mortality risks, the diabetic status may be a confounding factor.

Severely elevated glucose levels were significantly associated with MACEs compared with mildly to moderately elevated glucose levels. Higher blood glucose levels at admission were associated with higher incidence of multivessel disease (7) and acute kidney injury (30, 34), larger infarct size (30), lower initial success rate of primary PCI (7), and worse post-PCI TIMI flow grades (31)—degree of flow filling the distal coronary bed. Most events in MACEs were driven by in-hospital mortality (30) and heart failure (34). We also found that this association between AH and MACEs became stronger when patients received PCI within 24 h rather than primary PCI (RR 2.78 vs. 2.07), and that AH had long-term effects associated with MACEs albeit less than short-term effects (1.95 vs. 2.34).

At long-term follow-up, we found that the risk of all-cause mortality associated with admission hyperglycemia was 0.97-, 0.73-, and 1.12-fold higher in the total population of patients with diabetes or without diabetes, respectively. Lower risk of death was found compared to short-term mortality, but the AH had an increased risk for patients without diabetes than those with diabetes (RR 2.12 vs. 1.73). Patients who underwent primary PCI had a lower risk of death than those who underwent PCI within 24 h (RR 1.74 vs. 2.16). We divided follow-up into 1 year, 1–3 years, and > 3 years to reduce the impact of death from pre-existing conditions and found that AH was an independent factor associated with mortality risk. However, whether AH was associated with long-term mortality risk appeared to be controversial. Hyperglycemia on admission was no longer independently associated with long-term mortality when controlling for hemodynamic parameters, namely, blood pressure, heart rate on admission, and angiographic findings (42). Other study suggested that AH might be a reflection of an acute phase when patients developed myocardial infarction with or without hemodynamic compromise (6). Furthermore, this effect subsided over time in those patients who were initially glucose intolerant (62). Moreover, the previous study found that severely elevated glucose levels (≥ 190 mg/dL) were significantly associated with mortality risk (7), and the risk was higher in patients with non-diabetes than in patients with diabetes (38). We thought among those hyperglycemic patients without diabetes, a subset of the population had undiagnosed diabetes, some were untreated diabetes, and some may have pre-diabetes. The severely elevated glucose levels indicate that these people may already have abnormal glycemic status and admission hyperglycemia is a chance finding when they present to hospitals for AMI. Our suggestions were supported by a study (38) which concluded that the mean fasting glucose level had a better prognostic outcome than admission fasting glucose. Therefore, we thought that admission hyperglycemia lacked predictive properties to late mortality risk. An important study (42) that gave the value of HbA1c on admission in patients with non-diabetic STEMI undergoing primary PCI suggested that HbA1c may improve risk assessment for long-term mortality risk because HbA1c reflected average blood glucose over the past 3 months level. We think that HbA1c may be suitable for assessing long-term mortality. In a linear dose–response analysis of mortality, we found a 4.1% higher risk of death in patients with non-DM compared with patients with DM (10.9% vs. 6.8%; per 18 dl/ml: 1 mmol/l) which was significantly lower than a previous study (38) that reported each 18 mg/dl increase in the mean fasting glucose level was associated with a 46.8% increased risk of mortality.

## Implications of the study and future research

Considering the robust and significant association between admission hyperglycemia and all-cause mortality risk shown in our study, glucose-lowering therapy in patients with severely elevated glucose levels may improve outcomes. Although admission hyperglycemia is usually asymptomatic, it represents a biomarker (4) of adverse outcomes and complications in AMI. Two intensive insulin therapy for critically ill patients (patients with recent surgery or with sepsis) showed that strict normalization of blood glucose levels (80–110 mg/dl) can reduce morbidity such as newly acquired kidney injury but not mortality (63) and would increase the rate of severe hypoglycemic events (64). However, we did not think the treatment between critically ill patients and patients with acute coronary ischemia was similar, because those participants were distinctly different patient populations and most participants included in our study had stable hemodynamics and homeostasis. The American Diabetes Association recommends that insulin therapy should be initiated for the treatment of persistent hyperglycemia starting at a threshold ≥ 180 mg/dl (5). When the insulin therapy starts, a target glucose range of 140–180 mg/dl is recommended for the majority of critically and non-critically ill patients (level of evidence A) (50). A study of lower blood glucose levels in patients with AMI after admission showed that the normalization of blood glucose in patients with AMI with AH was associated with better survival whether or not they received insulin therapy and that insulin therapy had no significant independent effect on mortality (65). We think that AMI setting is distinct from that of long-term care and patients can receive the rapid benefit of insulin therapy because of its glucose-lowering effect. The follow-up study of DIGAMI 1 trial has shown that intensified insulin-based glycemic control after AMI had a long-lasting effect on longevity (66). Consequently, we believe that insulin therapy for patients with AMI undergoing primary PCI can not only reduces comorbidities such as no-flow and failure of PCI but also improves survival. However, based on current guidelines, therapeutic threshold interventions for glucose-lowering therapy for STEMI > 200 mg/dl (9) and NSTE-ACS > 180 mg/dl (10) seem to be conservative. In our study, we found that an admission glucose level threshold > 150 mg/dl could double the risk of mortality in patients with non-DM. Therefore, if continuous glucose monitoring is provided to prevent hypoglycemia, we suggest that a more stringent target (110–140 mg/dl) may be appropriate (5). Although the association between admission hyperglycemia and long-term mortality seemed to be significant, several studies found that the mean fasting glucose level (38) and HbA1c (42) were better predictors. We suggest that the routine measurement of HbA1c in patients with AH without diabetes can identify patients with unrecognized diabetes or those at increased risk of developing diabetes in future (67) and be beneficial for secondary prevention of AMI.

Lifestyle changes or community mobilization may lead to a reduction in developing diabetes and play a key prevention aspect of cardiovascular disease (68, 69). Future studies should evaluate the relationship between insulin therapy and patient outcomes in patients with AMI in different glucose level groups based on RCT design, aiming to find better therapeutic targets.

## Strengths and limitations of the study

Some important limitations must be noted when interpreting the results. First, the reporting bias may exist in short-term mortality because the missing studies were on the left side of the contour-enhanced plots where results would be unfavorable to the hyperglycemia intervention and broadly in the area of non-significance (*P* > 0.1). Based on this finding, we did not further analyze the trim and fill method. Second, although we excluded studies of reperfusion therapy specifically in patients with AMI with thrombolysis, some studies still included patients with thrombolysis. Third, potential confounding by different reperfusion therapies must be considered. Furthermore, in subgroup analyses, only six studies were appropriately adjusted for adequate factors. The results might be influenced by the effect of the geographic region because the number of studies in Europe, North America, and Africa were limited. The same limitation was also found in the glucose level assessment because only three studies were assessed by fasting plasma glucose. Fourth, we did not perform subgroup analyses for long-term MACE as only four studies reported sufficient data. In addition, we defined a group as the DM group when patients with diabetes exceeded 35% of the total population, which may overestimate the effect of diabetics. We defined the second group in Chung et al. study (8) as the control arm, because only five patients were in their first group, and zero events were found. The result might be overvalued by zero events when we adjusted these data. Last, we used approximations in some of the included studies to estimate mean doses of glucose levels, which may have led to overestimation in dose–response results.

The present study also has several strengths. First, we gathered all the evidence relating to the association of admission hyperglycemia with all-cause mortality risk. We included 23 cohort studies with 47,177 participants, which enabled us to test the associations in different subgroups. Second, we performed several subgroup analyses based on follow-up duration, diabetes status, type of AMI, time to PCI, geographical region, adequate adjustments, study quality, and glucose level assessment. Third, we performed linear dose–response analyses for the association between AH and all-cause mortality in patients with AMI with or without diabetes, the first dose–response meta-analysis on this topic. Finally, we found that for admission hyperglycemia, patients with non-diabetes had a higher risk than patients with diabetes and a lower cut-off threshold for intervention.

## Conclusion

The present meta-analysis of 23 cohort studies indicated that admission hyperglycemia was significantly associated with higher all-cause mortality risk. Moreover, caution was required when we used the findings that admission hyperglycemia had a long-term risk of all-cause mortality because many participants may already have unrecognized diabetes, impaired glucose tolerance, and pre-diabetes.

Our results suggested that lower intervention cut-off (> 150 mg/dl) and more stringent targets (110–140 mg/dl) for admission hyperglycemia may be more appropriate than current guideline recommendations. However, the analysis of aggregate data has led to the overestimation of relations, and several methodological approximations would have affected the analyses.

## Data availability statement

The original contributions presented in this study are included in the article/Supplementary material, further inquiries can be directed to the corresponding author/s.

## Author contributions

S-YC, H-W, J-HW, and X-CD contributed to conception and design of the study, acquisition of data, made the GRADE assessment (J-HW and X-CD received training), and final approval of the version to be published. All authors contributed to the article and approved the submitted version.
