# Supplementary material for: Association of admission hyperglycemia and all-cause mortality in acute myocardial infarction with percutaneous coronary intervention: A dose–response meta-analysis
Source: Front Cardiovasc Med. 2022 Sep 12;9:932716. doi: 10.3389/fcvm.2022.932716 (PMC9510712; doi:10.3389/fcvm.2022.932716)
Supplement: Supplementary file 1 [file Data_Sheet_1.docx]

**Supplemental file 1. Literature search for PubMed and EMBASE**

**PubMed**

| #1 | hyperglycemia [MeSH Terms] |
| --- | --- |
| #2 | "hyperglycaemia"[Title/Abstract] |
| #3 | "hyperglycemias"[Title/Abstract] |
| #4 | "hyperglycaemias"[Title/Abstract] |
| #5 | "blood glucose"[Title/Abstract] |
| #6 | "plasma glucose"[Title/Abstract] |
| #7 | "hyperglycemic"[Title/Abstract] |
| #8 | #1 OR #2 OR #3 OR #4 OR #5 OR #6 OR #7 |
| #9 | myocardial infarction [MeSH Terms] |
| #10 | myocardial infarction*[Title/Abstract] |
| #11 | STEMI[Title/Abstract] |
| #12 | #9 OR #10 OR #11 |
| #13 | primary angioplasty [Title/Abstract] |
| #14 | percutaneous coronary intervention*[Title/Abstract] |
| #15 | coronary intervention*[Title/Abstract] |
| #16 | PCI[Title/Abstract] |
| #17 | #13 OR #14 OR #15 OR #16 |
| #18 | #8 AND #12 AND #17 |

**Supplemental file 2. Characteristics of Included Studies**

| Author, Year | Country or Region | Population | Glucose measurement | AH Category  (mg/dl) | Sample Size | Hyperglycemia [n (%)] | Confounding factors | Outcomes |
| --- | --- | --- | --- | --- | --- | --- | --- | --- |
| Straumann et al. 2005 | Switzerland | AMI patients with or without DM treated with primary or rescue PCI | Random plasma glucose | Group I (<140)  Group II (140 -198)  Group III (> 198) | 978 | 656 (67.1) | - | 30-day and long-term mortality |
| Worthley et al. 2007 | Canada | STEMI patients with or without DM treated with primary PCI | Random plasma glucose | Group I (≤119)  Group II (120-140)  Group III (141-180)  Group IV (≥181) | 980 | 722 (73.7) | Age, gender, cardiogenic shock, and TIMI flow post procedure | In-hospital mortality |
| Lavi et al. 2007 | Israel | STEMI patients with or without DM treated with primary PCI | Fasting plasma glucose | Group I (91-109)  Group II (136-183)  Group III (132-209)-diabetes | 431 | 207 (48.0) | Age, gender, previous infarction, history of hypertension, time from symptom onset to first balloon inflation, infarct location, Killip class on admission, admission heart rate, admission blood pressure, presence of anterior infarction, left ventricular function by echocardiography, creatinine levels, baseline and final thrombolysis in myocardial infarction flow | In-hospital and long-term mortality |
| Ishihara et al. 2007 | Japan | AMI patients with or without DM treated with primary PCI | Random plasma glucose | Group I (<200)  Group II (≥200) | 802 | 261 (32) | age, gender, hypertension, current smoking, previous myocardial infarction, Killip class, anterior location, time to angiography, and primary coronary intervention | 30-day and long-term mortality |
| Kosiborod et al. 2008 | USA | AMI patients with or without DM treated with PCI | Random plasma glucose | Group I (<110)  Group II (110-140)  Group III (140-170)  Group IV (170-200)  Group V (≥200) | 16871 | 13501 (80) | - | In-hospital mortality |
| Usami et al. 2009 | Japan | STEMI patients without DM treated with PCI within 24 h after the onset | Random plasma glucose | Group I (<200)  Group II (≥200) | 2433 | 231 (9.5) | Age, male, BMI, Killip class, multivessel disease, intracoronary thrombectomy | 30-day mortality |
| Aronson et al. 2009 | Israel | AMI patients with or without DM treated with PCI, thrombolysis and coronary revascularization | Fasting plasma glucose | Group I (<100)  Group II (100-125)  Group III (≥126) | 1467 | 876 (59.7) | GRACE risk score | long-term mortality |
| Marenzi et al. 2010 | Italy | STEMI patients with or without DM treated with primary PCI | Random plasma glucose | Group I (≤198)  Group II (>198) | 780 | 148 (19.0) | AH, eGFR, DM, and contrast volume | In-hospital mortality  and MACEs |
| Timmer et al.  2011 | Netherlands | STEMI patients without known  diabetes mellitus treated with primary PCI | Random plasma glucose | Group I (≤124)  Group II (125-145)  Group III (145-171)  Group IV (≥172) | 4132^$^ | 3142 (75.0) | - | 30-day and long-term mortality |
| Hoebers et al. 2012 | Netherlands | STEMI patients with or without DM treated with primary PCI | Random plasma glucose | Group I (<140)  Group II (140 -198)  Group III (>198) | 1646 | 899 (54.6) | glucose level at admission, age,  male gender, body mass index, history of DM, hypertension, smoking status, hypercholesterolemia, previous myocardial infarction, shock, time to treatment (symptom onset to first balloon inflation), left anterior descending coronary artery–related myocardial infarction, and multivessel disease | 30-day and long-term Mortality |
| Ekmekci et al. 2013 | Turkey | STEMI patients without diabetes mellitus treated with primary PCI | Random plasma glucose | Group I (<118)  Group II (118 -145)  Group III (> 145) | 503 | 337 (67.0) | - | In-hospital mortality  and MACEs |
| Planer et al. 2013 | USA | STEMI patients with or without DM treated with primary PCI | Random plasma glucose | Group I (≤122.4)  Group II (122.4-156)  Group III (>156) | 3405 | 2269 (66.6) | age, gender, known diabetes at baseline, history of congestive heart failure, smoking history, baseline creatinine and hemoglobin levels, and left‐ventricular ejection fraction | In-hospital and long-term mortality  and MACEs |
| Chen et al. 2014 | China | STEMI patients with or without DM treated with primary PCI | Random plasma glucose | Group I (<100)  Group II (100-139)  Group III (140-189)  Group IV (190-249)  Group V (≥250) | 959 | 417 (43.4) | - | In-hospital and long-term mortality  and MACEs |
| Dharma et al. 2019 | Indonesia | STEMI patients with or without DM treated with primary PCI | Random plasma glucose | Group I (<169)  Group II (≥169) | 856 | 307 (35.9) | - | In-hospital and long-term mortality |
| Khalfallah et al.  2019 | Egypt | STEMI patients without DM treated with primary PCI | Random plasma glucose | Group I (96.9-128.3)  Group II (188-256.2) | 660 | 111 (16.8) | - | 90-day mortality and MACEs |
| Chung et al.  2020 | Korea | AMI patients with or without DM treated with PCI | Random plasma glucose | Group I (≤80)  Group II (81-140)  Group III (141-200)  Group IV (201-260)  Group V (≥261) | 1593 | 954 (59.9) | - | 30-day mortality |
| Demarchi et al.  2020 | Italy | STEMI patients with or without DM treated with primary PCI | Random plasma glucose | Group I (≤198)  Group II (>198) | 2958 | 488 (16.5) | age, anterior MI, Killip class≥2, chronic kidney disease, baseline Hb, admittance heart rate, ST Resolution, previous MI, hypertension and TIMI Flow Grade | long-term Mortality |
| Shahid et al.  2020 | Pakistan | STEMI patients with or without DM treated with primary PCI | Random plasma glucose | Group I (≤140)  Group II (>140) | 256 | 96 (37.5) | - | In-hospital mortality |
| Wang et al.  2020 | China | STEMI patients without DM treated with primary PCI | Fasting plasma glucose | Group I (≤109.8)  Group II (>109.8) | 623 | 161 (25.8) | - | 30-day mortality |
| Li et al.  2020 | China | STEMI patients with or without DM treated with primary PCI | Random plasma glucose | Group I [102 (91-118.8)]  Group II [120 (110.7-143.5)]  Group III [200 (141.3-247.7)] | 350 | 112 (32) | - | long-term Mortality and MACEs |
| Cui et al.  2021 | China | AMI patients with or without DM treated with PCI | Fasting plasma glucose | Non-DM Group I (<122)  Non-DM Group II (≥122)  DM Group III (<266)  DM Group IV (≥266) | 1228 | 756 (61.6) |  | In-hospital and long-term Mortality and  MACEs |
| Liu et al.  2021 | China | NST-ACS patients without DM treated with PCI | Random plasma glucose | Group I (<140)  Group II (140-200)  Group III (≥200) | 498 | 374 (75.1) | - | In-hospital mortality  and MACEs |
| Ferreira et al. 2021 | Portugal | AMI patients with or without DM treated with PCI | Random plasma glucose | Group I (≤143)  Group II (>143)  Group III (≤213)  Group IV (>213) | 2768 | 1344 (48.6) | Age, gender, active smoking, previous AMI, GRACE score, hypertension, previous stroke/TIA and left-ventricular ejection fraction, dyslipidemia, CRP, LDL, serum creatinine, Troponin I, and Killip–Kimball class ≥ 2 at admission, Beta-blocker and ACE-inhibitor during hospitalization | long-term Mortality |

**Supplemental file 3. Quality Assessment of the included studies**

| **Study** | **Selection** | | | | **Comparability** | **Outcome** | | | **Total**  **Score** |
| --- | --- | --- | --- | --- | --- | --- | --- | --- | --- |
|  | **Exposed**  **Cohort** | **Nonexposed**  **Cohort** | **Ascertainment**  **of Exposure** | **Outcome**  **of Interest** |  | **Assessment**  **of Outcome** | **Length of**  **Follow-up** | **Adequacy**  **of Follow-up** |  |
| Straumann et al. 2005 | ★ | ★ | ★ | ★ | ★★ | - | ★ | ★ | Good (8) |
| Worthley et al. 2007 | ★ | ★ | ★ | ★ | ★★ | ★ | - | ★ | Good (8) |
| Lavi et al. 2007 | ★ | ★ | ★ | ★ | ★★ | - | - | ★ | Good (7) |
| Ishihara et al. 2007 | ★ | ★ | ★ | ★ | ★★ | ★ | ★ | ★ | Good (9) |
| Kosiborod et al. 2008 | ★ | ★ | ★ | ★ | ★★ | - | ★ | ★ | Good (8) |
| Usami et al. 2009 | ★ | ★ | ★ | ★ | ★★ | ★ | - | ★ | Good (8) |
| Aronson et al. 2009 | ★ | ★ | ★ | ★ | ★★ | - | ★ | ★ | Good (8) |
| Marenzi et al. 2010 | ★ | ★ | ★ | ★ | ★★ | ★ | - | ★ | Good (8) |
| Timmer et al. 2011 | ★ | ★ | ★ | ★ | ★★ | - | ★ | ★ | Good (8) |
| Hoebers et al. 2012 | ★ | ★ | ★ | ★ | ★★ | ★ | ★ | ★ | Good (9) |
| Ekmekci et al. 2013 | ★ | ★ | ★ | ★ | ★★ | - | - | ★ | Good (7) |
| Planer et al. 2013 | ★ | ★ | ★ | ★ | ★★ | ★ | ★ | ★ | Good (9) |
| Chen et al. 2014 | ★ | ★ | ★ | ★ | ★★ | - | ★ | ★ | Good (8) |
| Dharma et al. 2019 | ★ | ★ | ★ | ★ | ★★ | - | ★ | ★ | Good (8) |
| Kalińczuk et al. 2017 | ★ | ★ | ★ | ★ | ★★ | - | - | ★ | Good (7) |
| Chung et al. 2020 | ★ | ★ | ★ | ★ | ★★ | - | - | ★ | Good (7) |
| Demarchi et al. 2020 | ★ | ★ | ★ | ★ | ★★ | ★ | - | ★ | Good (8) |
| Shahid et al. 2020 | ★ | ★ | ★ | ★ | ★ | - | - | ★ | Fair (6) |
| Wang et al. 2020 | ★ | ★ | ★ | ★ | ★★ | - | - | ★ | Good (7) |
| Li et al. 2020 | ★ | ★ | ★ | ★ | ★★ | - | ★ | ★ | Good (8) |
| Cui et al. 2021 | ★ | ★ | ★ | ★ | ★★ | - | ★ | ★ | Good (8) |
| Liu et al. 2021 | ★ | ★ | ★ | ★ | ★★ | - | - | ★ | Good (7) |
| Ferreira et al. 2021 | ★ | ★ | ★ | ★ | ★★ | ★ | ★ | ★ | Good (9) |

**Supplemental file 4a. Sensitivity Analysis of Short-term all-cause mortality**

| Omitting study | RR (95%CI) | *I*^2^(%) |
| --- | --- | --- |
| Straumann et al (2005) | 2.99 (2.33-3.85) | 74.2 |
| Worthley et al (2007) | 3.06 (2.37-3.93) | 75.3 |
| Lavi et al (2007) | 3.06 (2.37-3.95) | 75.5 |
| Ishihara et al (2007) | 3.28 (2.51-4.28) | 75.1 |
| Kosiborod et al (2008) | 3.25 (2.58-4.10) | 56.4 |
| Usami et al (2009) | 2.93 (2.30-3.73) | 70 |
| Marenzi et al (2010) | 3.11 (2.39-4.05) | 75.9 |
| Timmer et al (2011) | 3.20 (2.45-4.18) | 76.2 |
| Hoebers et al (2012) | 3.05 (2.35-3.96) | 74.6 |
| Planer et al (2013) | 3.02 (2.34-3.91) | 74.8 |
| Ekmekci et al (2013) | 3.09 (2.40-3.98) | 75.8 |
| Chen et al (2014) | 3.29 (2.55-4.25) | 74.4 |
| Dharma et al (2019) | 3.14 (2.41-4.10) | 76.1 |
| Khalfallah et al (2019) | 3.18 (2.44-4.14) | 76.2 |
| Chung et al (2020) | 3.13 (2.40-4.08) | 76.1 |
| Shahid et al (2020) | 3.12 (2.41-4.06) | 76.1 |
| Wang et al (2020) | 3.07 (2.37-3.97) | 75.7 |
| Cui et al (2021) | 3.19 (2.42-4.20) | 76.2 |
| Liu et al (2021) | 3.10 (2.36-4.07) | 72.8 |

**Supplemental file 4b. Sensitivity Analysis of Short-term MACEs**

| Omitting study | RR (95%CI) | *I*^2^(%) |
| --- | --- | --- |
| Lavi et al (2007) | 2.32 (1.72-3.14) | 85.9 |
| Marenzi et al (2010) | 2.35 (1.71-3.23) | 85.9 |
| Ekmekci et al (2013) | 2.29 (1.71-3.08) | 85.7 |
| Planer et al (2013) | 2.30 (1.69-3.12) | 85.6 |
| Chen et al (2014) | 2.54 (1.95-3.31) | 80.7 |
| Usami et al (2009) | 2.07 (1.72-2.49) | 59 |
| Khalfallah et al (2019) | 2.44 (1.80-3.31) | 84.4 |
| Wang et al (2020) | 2.40 (1.75-3.29) | 85.5 |
| Cui et al (2021) | 2.34 (1.72-3.19) | 85.9 |
| Liu et al (2021) | 2.38 (1.74-3.24) | 85.9 |

**Supplemental file 4c. Sensitivity Analysis of Long-term all-cause mortality**

| Omitting study | RR (95%CI) | *I*^2^(%) |
| --- | --- | --- |
| Straumann et al (2005) | 1.95 (1.57-2.43) | 74.1 |
| Ishihara et al (2007) | 2.10 (1.78-2.48) | 53.4 |
| Aronson et al (2009) | 1.98 (1.58-2.48) | 74.4 |
| Timmer et al (2011) | 2.05 (1.67-2.53) | 70.8 |
| Hoebers et al (2012) | 1.89 (1.54-2.32) | 70.1 |
| Planer et al (2013) | 1.99 (1.59-2.49) | 74.5 |
| Chen et al (2014) | 2 (1.62-2.47) | 74.1 |
| Dharma et al (2019) | 1.97 (1.59-2.44) | 74.5 |
| Li et al (2020) | 1.88 (1.56-2.27) | 67.7 |
| Demarchi et al (2020) | 1.99 (1.59-2.48) | 74.5 |
| Cui et al (2021) | 1.92 (1.55-2.37) | 71 |
| Ferreira et al (2021) | 1.98 (1.57-2.50) | 74.5 |

**Supplemental file 4d. Sensitivity Analysis of Long-term MACEs**

| Omitting study | RR (95%CI) | I^2^(%) |
| --- | --- | --- |
| Planer et al (2013) | 2.38 (1.36-4.19) | 73.2 |
| Chen et al (2014) | 2.10 (1.20-3.67) | 92.9 |
| Li et al (2020) | 1.96 (0.86-4.48) | 88.7 |
| Cui et al (2021) | 1.95 (1.21-3.14) | 89.3 |

**Supplemental file 5. Contour-enhanced funnel plot of all-cause mortality in AMI patients associated with admission hyperglycemia**

**
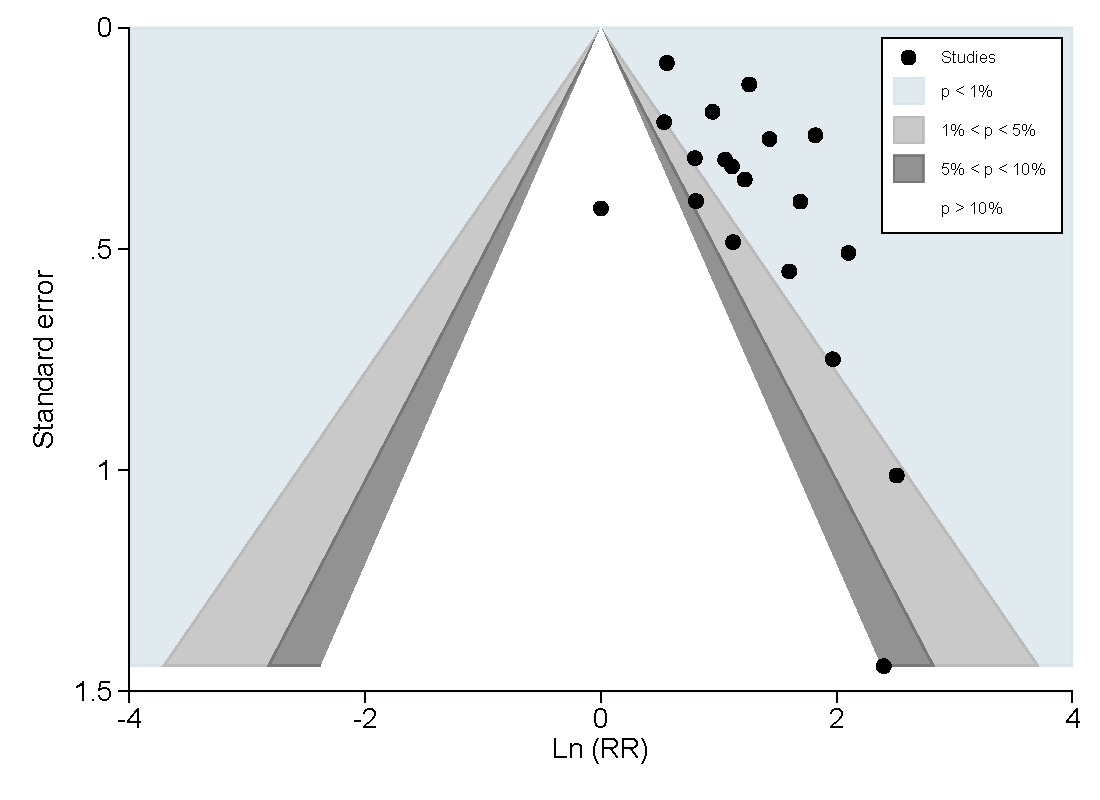
**

**Supplemental file 6. Forrest plot of admission hyperglycemia associated with the short-term MACEs**

**
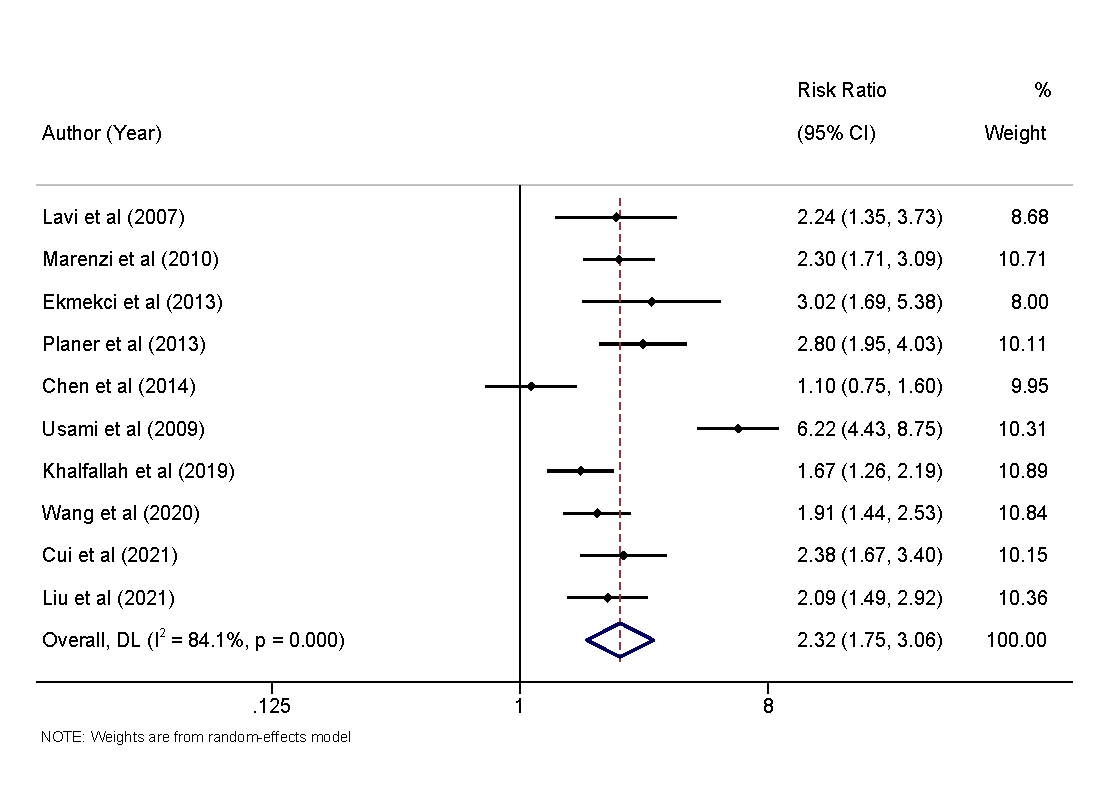
**

**Supplemental file 7. Forrest plot of admission hyperglycemia associated with the long-term MACEs**

**
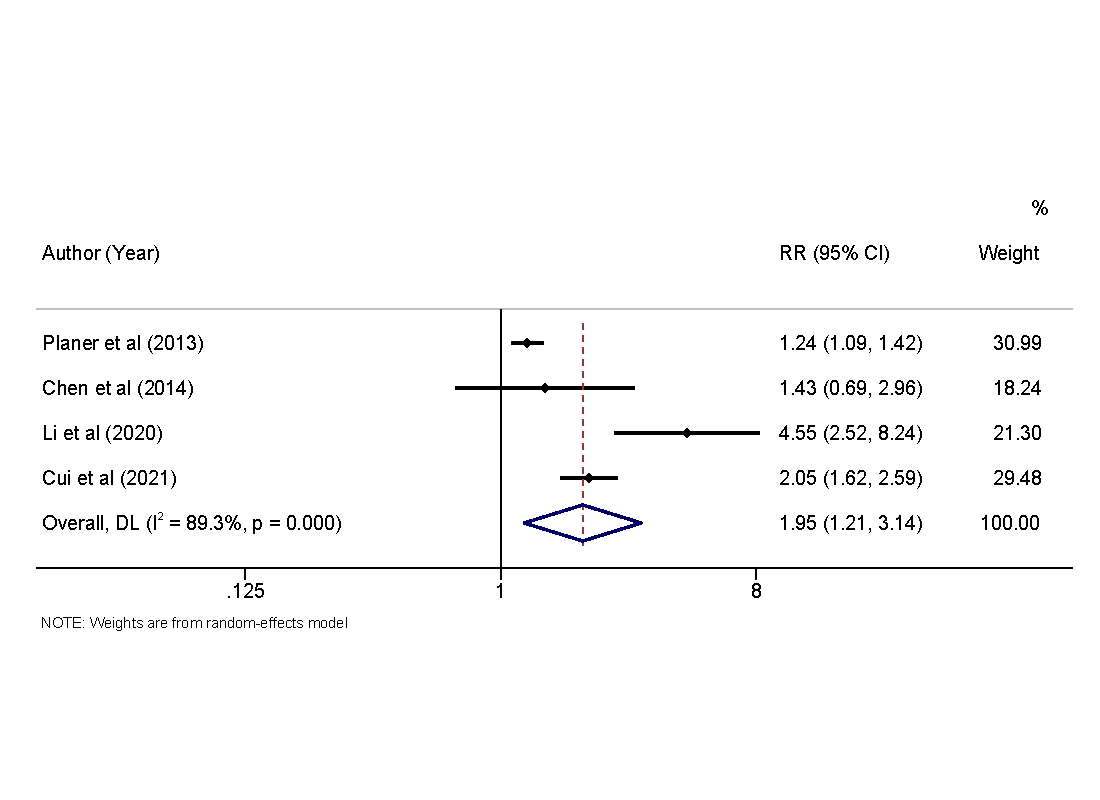
**

**Supplemental file 8. GRADE Evidence Profile of Admission hyperglycemia vs Placebo with Acute Myocardial Infarction Included in Cohort Studies**

| Outcomes | No. of Studies | No. of Patients | Follow-up | Serious Risk of Bias | I^2^ (%) | Serious Indirectness or Imprecision | P value for Publication Bias | Relative Risk (95%CI) | Quality of Evidence |
| --- | --- | --- | --- | --- | --- | --- | --- | --- | --- |
|  |  |  |  |  |  |  |  |  |  |
| short-term all-cause mortality | 19 | 39629 | 0 to 3 months | No | 74.8 | serious^a^ | 0.015 | **3.12**  (2.42 to 4.02) | Moderate |
| short-term MACEs | 10 | 11520 | 0 to 3 months | No | 84.2 | serious^b,c^ | 0.55 | **2.34**  (1.77 to 3.09) | Low |
| long-term all-cause mortality | 12 | - | ≥1 year | No | 71.9 | Serious^d^ | 0.56 | **1.97**  (1.61 to 2.41) | Moderate |
| long-term MACEs | 4 | 7171 | ≥1 year | No | 89.3 | Serious^b,c,e^ | 0.30 | **1.95**  (1.21 to 3.14) | Very low |

a. All included studies were cohort studies

b. The acute myocardial infarction has the complications which were some of MACEs.

c. Most of included studies did not use adjustment model

d. participants with pre-existing diseases are more likely to experience death in the early years of follow-up.

e. from the sensitivity analysis, the result was not robust.
